# Group IIA Secretory Phospholipase A_2_ Predicts Graft Failure and Mortality in Renal Transplant Recipients by Mediating Decreased Kidney Function

**DOI:** 10.3390/jcm9051282

**Published:** 2020-04-29

**Authors:** Wijtske Annema, Jan Freark de Boer, Arne Dikkers, Lidiya G. Dimova, Markus van der Giet, Stephan J.L. Bakker, Uwe J.F. Tietge

**Affiliations:** 1 Department of Pediatrics, University of Groningen, University Medical Center Groningen, 9713 GZ Groningen, The Netherlands; 2 Medizinische Klinik IV—Nephrology, Charite—Campus Benjamin Franklin, 12203 Berlin, Germany; 3 Department of Internal Medicine, Division of Nephrology, University of Groningen, University Medical Center Groningen, 9713 GZ Groningen, The Netherlands; 4 Division of Clinical Chemistry, Department of Laboratory Medicine, Karolinska Institutet, 141 86 Stockholm, Sweden; 5 Clinical Chemistry, Karolinska University Laboratory, Karolinska University Hospital, 141 86 Stockholm, Sweden

**Keywords:** transplantation, phospholipase, prospective, kidney function, mortality, cohort study, biomarker

## Abstract

The acute phase protein group IIA secretory phospholipase A_2_ (sPLA_2_-IIA) has intrinsic proatherosclerotic properties. The present prospective cohort study investigated whether plasma sPLA_2_-IIA associates with graft failure, cardiovascular, and all-cause mortality in renal transplant recipients (RTRs), patients with accelerated atherosclerosis formation both systemically and within the graft. In 511 RTRs from a single academic center with stable graft function >1 year, baseline plasma sPLA_2_-IIA was determined by ELISA. Primary end points were death-censored graft failure and mortality (median follow-up, 7.0 years). Baseline sPLA_2_-IIA was higher in RTRs than in healthy controls (median 384 ng/dL (range 86–6951) vs. 185 ng/dL (range 104–271), *p* < 0.001). Kaplan–Meier analysis demonstrated increased risk for graft failure (*p* = 0.002), as well as cardiovascular (*p* < 0.001) and all-cause mortality (*p* < 0.001), with increasing sPLA_2_-IIA quartiles. Cox regression showed strong associations of sPLA_2_-IIA with increased risks of graft failure (hazard ratio (HR) = 1.42 (1.11–1.83), *p* = 0.006), as well as cardiovascular (HR = 1.48 (1.18−1.85), *p* = 0.001) and all-cause mortality (HR = 1.39 (1.17−1.64), *p* < 0.001), dependent on parameters of kidney function. Renal function during follow-up declined faster in RTRs with higher baseline sPLA_2_-IIA levels. In RTRs, sPLA_2_-IIA is a significant predictive biomarker for chronic graft failure, as well as overall and cardiovascular disease mortality dependent on kidney function. This dependency is conceivably explained by sPLA_2_-IIA impacting negatively on kidney function.

## 1. Introduction

For end-stage renal disease (ESRD) patients, kidney transplantation is generally accepted to be the treatment modality resulting in the highest gain in quality of life and in the lowest morbidity as well as mortality rates. Although kidney transplantation decreases the excessive age-adjusted increase in overall and specifically cardiovascular mortality associated with ESRD, renal transplant recipients (RTRs) still exhibit 4–6-fold higher rates of cardiovascular disease mortality compared with the general population [1]. It is well established that an increased inflammatory load constitutes a major contributing factor to such observations [2,3]. In addition, atherosclerosis within the kidney graft resulting in transplant vasculopathy with subsequent chronic graft failure represents a significant clinical problem for RTRs [4,5]. It has been noted that, already, after five years, approximately 50% of RTRs develop transplant vasculopathy and that 90% develop it 10 years after transplantation [4]. Vascular alterations resemble classical atherosclerotic lesions with a significant inflammatory component and lead to progressive transplant fibrosis and successive structural loss of organ function, ultimately requiring re-transplantation or return to dialysis [5,6,7]. In contrast to the clinical significance of the problem, specific therapeutic options to treat transplant vasculopathy are, however, limited [4,5].

The group IIA secretory phospholipase A_2_ (sPLA_2_-IIA) is an acute phase protein increasingly recognized as a biomarker of cardiovascular disease with intrinsic pro-atherosclerotic biological activity [8,9,10]. Higher amounts of sPLA_2_-IIA are found in atherosclerotic lesions, where the enzyme is mainly expressed in macrophages and vascular smooth muscle cells [11,12,13]. In mouse models, sPLA_2_-IIA has been shown to link the dyslipidemia of inflammatory responses to oxidative stress, and subsequently to increased formation of atherosclerotic lesions [14,15,16,17,18,19]. In humans, circulating levels of sPLA_2_-IIA are cross-sectionally associated with the presence of atherosclerotic cardiovascular disease in the general population [20,21,22], and have also been shown to serve as predictor for the future occurrence of cardiovascular disease events [20,22,23,24,25,26]. In cross-sectional work, we have previously reported that plasma sPLA_2_-IIA levels are substantially elevated in ESRD patients on hemodialysis [27]. However, prospective data on the impact of sPLA_2_-IIA on clinically meaningful cardiovascular disease outcomes in patient groups with impaired kidney function or ESRD have not been reported. Likewise, circulating sPLA_2_-IIA levels and the potential prospective relationship to clinical outcomes of atherosclerosis in RTRs have, to the best of our knowledge, not been a subject of study thus far.

Therefore, the aim of this prospective study was to investigate whether circulating levels of sPLA_2_-IIA are associated with future graft failure and all-cause as well as cardiovascular mortality in RTR.

## 2. Experimental Section

### 2.1. Study Design and Patients

With respect to RTRs, we carried out a prospective longitudinal cohort study, where every adult RTR patient seen in the nephrological outpatient clinic of the University Medical Center Groningen in the time span August 2001 to July 2003 was in principle eligible to participate when a stable graft function was present for at least one year (median time between transplantation and inclusion 6.0 years, for detailed distribution, please see Table 1). In the outpatient clinic, RTRs are subjected to a continuous surveillance system where visits occur with a declining frequency, as recommended by the American Transplantation Society guidelines. This means that, immediately after hospital discharge, patients are seen twice a week, while the frequency declines to twice a year in the stable long-term course after transplantation [28]. In total, 847 RTRs were eligible to participate. Out of these, 606 signed the informed written consent form, and subsequently participated in the study. The group not choosing to participate was essentially identical to the group that chose to be part of the study with respect to age, sex, kidney function (judged by serum creatinine, creatinine clearance, and proteinuria), as well as body mass index (BMI). If a patient initially presented with fever or displayed otherwise signs of an ongoing infection (e.g., symptoms compatible with upper respiratory tract or urinary tract infections), the baseline visit was postponed until the symptoms were not present any longer. Exclusion criteria were overt congestive heart failure or cancer other than cured skin cancer. Plasma sPLA_2_-IIA levels were determined in 511 recipients. Full details of the study design have been published previously [29]. The study protocol was approved by the Institutional (UMCG) Review Board in 2001 (METc 2001/039), and was in compliance with the Declaration of Helsinki.

In order to place measurements of plasma sPLA_2_-IIA into a clinical context, we additionally investigated a group of ESRD patients (*n* = 60) as well as healthy controls that were matched by age and sex (*n* = 30) (clinical characteristics given in Supplemental Table S1). ESRD patients and controls were clinically stable and it was confirmed that they had not experienced an infection or another intercurrent illness in a time frame of at least three months before blood draw. ERSD patients had no residual kidney function. Blood draws in the ESRD group were carried out ahead of a regular hemodialysis session. All patients gave informed consent. The medical ethics committee at the Charité in Berlin approved the study.

### 2.2. End Points of the Study

The study had the following primary end-points, death-censored graft failure and cardiovascular-specific as well as overall mortality. The end-point death-censored graft failure was reached when RTRs returned to therapy with dialysis or were re-transplanted. The UMCG has a continuous system of patient surveillance implemented in the outpatient clinic to ensure that all clinical information on the patients is current and that causes of death are known and continuously updated. If a patient status is unclear, the responsible referring doctors are contacted. To code causes of death, the International Classification of Diseases in its 9th revision (ICD-9) was used [30]. As definition of cardiovascular death, ICD-9 codes 410 to 447 were applied. Death-censored graft failure and mortality were recorded until May 2009. No losses during follow-up occurred.

### 2.3. Renal Transplant Characteristics

The Groningen Renal Transplant Database holds information on all RTRs receiving grafts at the University Medical Center Groningen since 1968, including dialysis history. Relevant transplant characteristics, such as age, sex, date of transplantation, and donor demographics, were obtained using this database. Smoking status and history of cardiovascular disease were recorded with a self-report questionnaire when RTRs were included in the study. A previous myocardial infarction, transient ischemic attack, or cerebrovascular accident constituted a positive history of cardiovascular disease.

The following standard immunosuppressive regimens were used: from 1968 until 1989, prednisolone and azathioprine (100 mg/day); from January 1989 to February 1993, a standard formulation of cyclosporine (Sandimmun, Novartis Pharma B.V. Arnhem, The Netherlands; 10 mg/kg; trough levels of 175 to 200 μg/L for the months 0–3, 150 μg/L in months 3–12 post-transplant, and 100 μg/L after the first year) combined with prednisolone (starting dose 20 mg/day, which is lowered rapidly to 10 mg/day); from March 1993 to May 1997, a microemulsion formulation of cylosporine (Neoral, Novartis Pharma B.V., Arnhem, The Netherlands; 10 mg/kg body weight, trough levels as detailed above) and prednisolone; from May 1997 until present, mycophenolate mofetil (CellCept, Roche B.V., Woerden, The Netherlands; dosage 2 g/day) was added. Current medication use and changes in prednisolone dosage were taken from the respective medical records of the participants.

### 2.4. Measurements and Definitions

BMI was calculated as weight (kilogram) divided by length (meters squared), and used as an overall indicator of obesity. Waist circumference was determined on bare skin midway between the iliac crest and 10th rib with a tape measure. Blood pressure recordings were carried out in supine position after 6 min of rest using the average of three automated measurements taken in intervals of 3 min (Omron M4; Omron Europe).

Before visiting the outpatient clinic, patients collected urine for 24 h. Blood samples were drawn following an 8 to 12 h overnight fast, including no medication intake, which also applied for anti-hypertensive and glucose lowering drugs. Blood was drawn at the time of inclusion into the study and plasma and serum samples were continuously stored during the whole follow-up period at −80 °C; for the measurement of sPLA_2_-IIA, a previously unthawed aliquot was used. Total cholesterol levels were measured with the cholesterol oxidase phenol 4-aminoantipyrine peroxidase (CHOD-PAP) method (MEGA AU 510; Merck Diagnostica, Darmstadt, Germany), high-density lipoprotein (HDL) cholesterol with the CHOD-PAP method (Technikon RA-1000, Bayer Diagnostics B.V., Mijdrecht, The Netherlands), and low-density lipoprotein (LDL) cholesterol was calculated applying the Friedewald formula [31]. The glycerol-3-phosphate oxidase-phenol aminophenazone method was used for determining triglycerides, and the glucose-oxidase method (YSI 2300 Stat plus; Yellow Springs, OH, USA) for plasma glucose levels. Fasting serum insulin levels were measured by radioimmunoassay (DSL-1600; Diagnostic System Laboratories, Webster, TX, USA). The homeostasis model assessment (HOMA) was calculated according to the formula (glucose (mmol/L) × insulin (µU/mL))/22.5 [32]. HbA1c was determined by high performance liquid chromatography (VARIANTTM HbA1c Program with Bio-Rad CARIANT Hb Testing System, Biorad, Hercules, CA, USA). For measuring serum high-sensitivity C-reactive protein (hsCRP), an enzyme-linked immunosorbent assay was used as published [33]. Plasma sPLA_2_-IIA was assessed with an enzyme-linked immunosorbent assay (Cayman Chemical, Ann Arbor, MI, USA) according to the manufacturer’s instructions. For quantifying serum and urinary creatinine levels, a modified Jaffé method was applied (MEGA AU 510, Merck Diagnostica, Darmstadt, Germany). Renal allograft function was determined as creatinine clearance from 24 h urinary creatinine excretion and serum creatinine clearance. The glomerular filtration rate (GFR) was estimated with the CKD-EPI equation [34]. Urinary protein excretion was quantified with the Biuret reaction (MEGA AU 510; Merck Diagnostica, Darmstadt, Germany), the cut-off for proteinuria was a urinary protein excretion ≥0.5 g/24 h [28,35].

Diagnosis of the metabolic syndrome was made following the definition of the National Cholesterol Education Program Expert Panel (NCEP-ATP) recommendation [36] when three or more of the following components were present: (1) waist circumference >102 cm (men) or >88 cm (women); (2) serum triglycerides ≥1.70 mmol/L; (3) serum HDL-cholesterol <1.03 mmol/L (men) or <1.29 mmol/L (women); (iv) blood pressure ≥130/85 mmHg or use of anti-hypertensive medication; and (v) fasting plasma glucose ≥6.1 mmol/L or use of anti-diabetic medication. Following updated recommendations by the American Diabetes Association (ADA), a cut-off point ≥5.6 mmol/L was applied to define impaired fasting glucose [37]. A diagnosis of diabetes was established following ADA guidelines (fasting plasma glucose ≥7.0 mmol/L or HbA1c ≥6.5% or use of anti-diabetic medication (including insulin)) [38].

To assess changes in kidney function over time, the baseline kidney function at inclusion was related to subsequent kidney function measurements carried out at respective outpatient clinic visits until three years after baseline.

### 2.5. Statistical Analysis

Data analyses were carried out with the Statistical Package for the Social Sciences (SPSS) version 20 (SPSS Inc., Chicago, IL, USA) and GraphPad Prism version 5.0 (GraphPad Software Inc., San Diego, CA, USA). Normally distributed continuous variables are summarized using means ± standard deviations, whereas variables with a skewed distribution are given as medians (25th–75th percentiles). Categorical variables are expressed as numbers (percentages). Variables with a skewed distribution were log-transformed. Hazard ratios (HRs) are reported with 95% confidence interval (95% CI).

Patient characteristics were analyzed separately for sex-stratified quartiles of plasma sPLA_2_-IIA levels. Differences between the quartiles of sPLA_2_-IIA were tested for statistical significance with one-way analysis of variance (ANOVA) followed by Bonferroni post hoc test for normally distributed variables, with the Kruskal–Wallis test followed by Mann–Whitney U test for variables with a skewed distribution, and with the χ^2^ test for categorical variables.

Linear regression analysis was performed to evaluate the relationship between log-transformed plasma levels of hsCRP and log-transformed plasma levels of sPLA_2_-IIA. To analyze the association of plasma sPLA_2_-IIA levels with graft failure and mortality, sex-stratified quartiles of sPLA_2_-IIA were plotted in Kaplan–Meier analyses with log-rank test to assess significance between groups. Univariate and multivariate Cox proportional hazard regression was performed to investigate whether plasma levels of sPLA_2_-IIA are independently associated with graft failure, all-cause mortality, and cardiovascular mortality. For these analyses, plasma levels of sPLA_2_-IIA were first log-transformed to achieve normal distribution and entered in the model as a continuous variable. In the multivariate analyses, the associations of plasma sPLA_2_-IIA levels with graft failure, all-cause mortality, and cardiovascular mortality were adjusted for recipient age and sex (model 2); and further for serum creatinine (model 3); creatinine clearance (model 4); estimated glomerular filtration rate (eGFR) (model 5); urinary protein excretion (model 6); proteinuria (model 7); donor age and sex (model 8); living donor, dialysis vintage, and time between transplantation and inclusion (model 9); smoking (model 10); history of cardiovascular disease (model 11); log hsCRP (model 12); waist circumference, log triglycerides, and Framingham risk factors (systolic blood pressure, presence of diabetes, LDL-cholesterol, HDL-cholesterol, smoking) (model 13); and presence of diabetes, Hb1Ac, glucose, insulin, use of insulin, and use of anti-diabetic drugs (model 14). A two-sided *p*-value less than 0.05 was considered statistically significant.

## 3. Results

Circulating levels of sPLA_2_-IIA were measured in a total of 511 RTRs (mean age 51.7 ± 12.0 years, 54% men). Plasma levels of sPLA_2_-IIA in RTRs had a median value of 384 ng/dL (range 86 to 6951 ng/dL), which is significantly higher compared with controls from the general population (185 ng/dL (range 104–271 ng/dL); *p* < 0.001), although lower than in ESRD patients on hemodialysis (1053 ng/dL (range 458–2599 ng/dL); *p* < 0.001). Next, RTRs were divided into sex-stratified quartiles based on plasma levels of sPLA_2_-IIA. Baseline characteristics according to sex-stratified quartiles of sPLA_2_-IIA are presented in Table 1. Median values of sPLA_2_-IIA were 227.2 (196.1–248.3 ng/dL), 321.4 (290.7–356.5 ng/dL), 444.7 (402.0–504.3 ng/dL), and 757.4 (610.1–968.2 ng/dL) in the first, second, third, and fourth quartile, respectively. Patients in the upper quartile of sPLA_2_-IIA were older, were more frequently current smokers, used more diuretics, and had higher HbA1c percentages. However, the strongest and most consistent positive associations were found between plasma levels of sPLA_2_-IIA and conventional markers for renal allograft function, that is, serum creatinine, creatinine clearance, eGFR, urinary protein excretion, and proteinuria.

**Figure 1:**
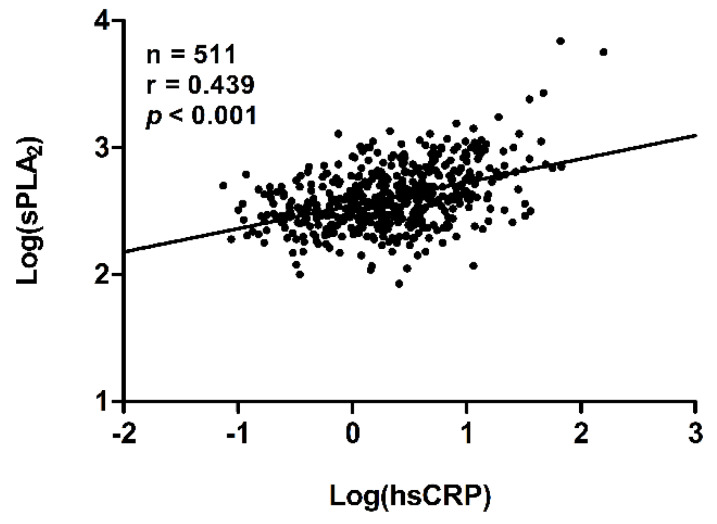
Correlation between plasma levels of log-transformed high sensitivity C-reactive protein (hsCRP) and log-transformed group IIA secretory phospholipase A_2_ (sPLA_2_). The regression line was fitted with linear regression. Pearson’s correlation coefficient is given.

Furthermore, hsCRP levels, a more general marker of inflammation, increased across quartiles of sPLA_2_-IIA. In line, univariate linear regression showed a strong positive correlation between log-transformed plasma levels of hsCRP and log-transformed plasma levels of sPLA_2_-IIA (r = 0.439, *p* < 0.001; Figure 1).

**Figure 2:**
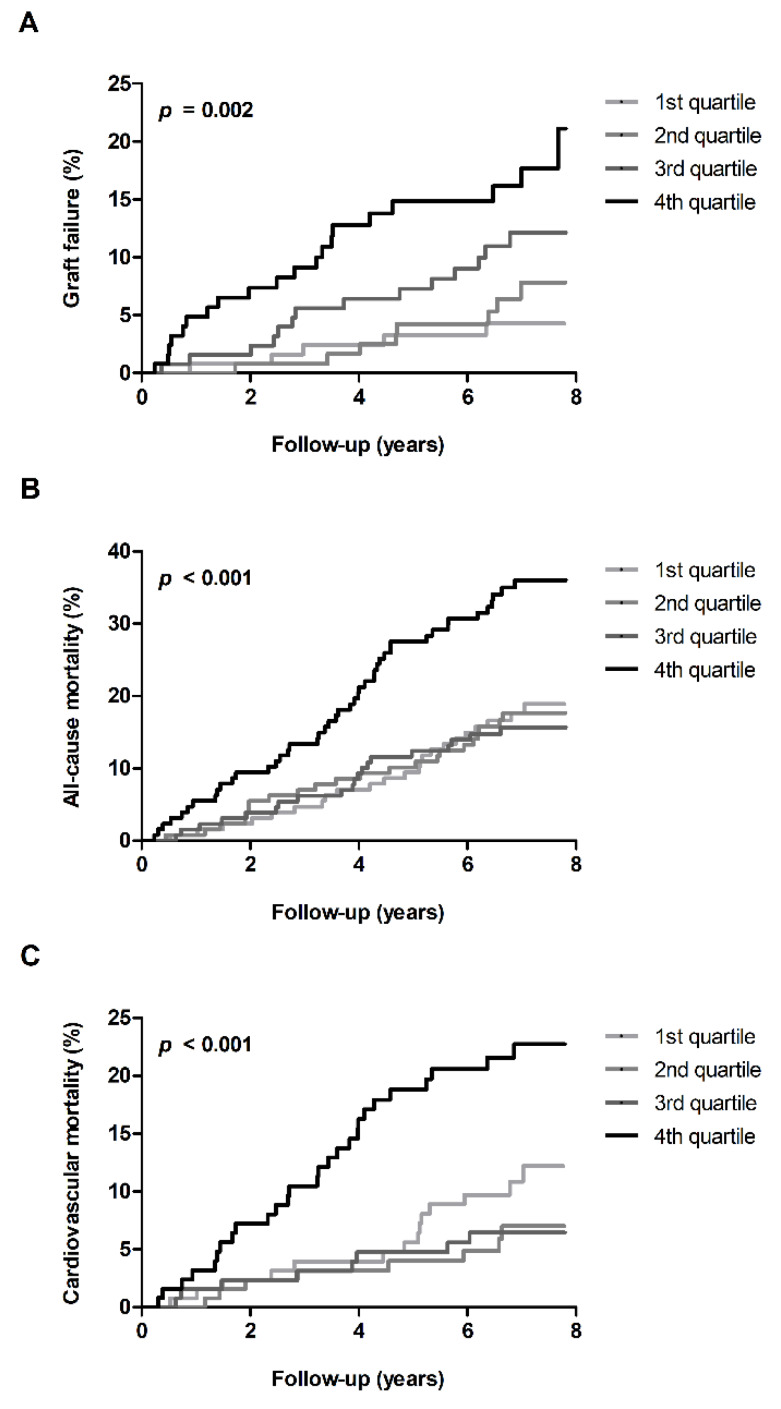
Kaplan–Meier curves of graft failure (**A**), all-cause mortality (**B**), and cardiovascular mortality (**C**) according to sex-stratified quartiles of plasma levels of group IIA secretory phospholipase A_2_ (sPLA_2_-IIA).

Next, we determined whether plasma levels of sPLA_2_-IIA were associated with future graft failure and mortality. During a median follow-up of 7.0 years (6.2–7.5), 9.2% (*n* = 47) of kidney grafts failed. We first used Kaplan–Meier analyses to compare graft failure across sex-stratified quartiles of plasma sPLA_2_-IIA levels. Incidence of graft failure was 5 (4%), 8 (6%), 14 (11%), and 20 (16%) according to increasing quartiles of sPLA_2_-IIA (log-rank test: *p* = 0.002; Figure 2A). Of 511 RTRs examined, 110 died during follow-up, with cardiovascular causes accounting for more than half (*n* = 57) of these deaths. Kaplan–Meier analyses revealed that all-cause mortality and cardiovascular mortality were significantly higher in RTRs in the highest quartile of plasma sPLA_2_-IIA compared with those in the three lower quartiles. RTRs in the upper sPLA_2_-IIA quartile had the highest incidence of all-cause mortality, 45 (35%), whereas the all-cause mortality was comparable in the three lower quartiles: 23 (18%) for the first, 22 (17%) for the second, and 20 (16%) for the third quartile (log-rank test: *p* < 0.001; Figure 2B). Similar results were observed for deaths of apparent cardiovascular origin. In the highest quartile of sPLA_2_-IIA, 27 (21%) died of cardiovascular disease, while the number of cardiovascular deaths was 14 (11%), 8 (6%), and 8 (6%) in the first, second, and third quartile, respectively (log-rank test: *p* < 0.001; Figure 2C).

Subsequently, univariate and multivariate Cox regression analyses were performed to calculate hazard ratios. The results of univariate Cox proportional hazard model analysis for graft failure demonstrated that higher plasma levels of sPLA_2_-IIA were associated with an increased risk of graft failure (hazard ratio (HR) = 1.42 (1.11–1.83), *p* = 0.006, model 1, Table 2). In multivariate analyses, adjustment for patient age and sex strengthened the association with graft failure (model 2). This link between circulating sPLA_2_-IIA and graft failure seemed to be largely explained by renal function, as the association disappeared after further adjustment for serum creatinine (model 3), for creatinine clearance (model 4), and for eGFR (model 5). On the other hand, adjustments for urinary protein excretion (model 6); for proteinuria (model 7); for donor age and sex (model 8); for living donor, dialysis vintage, and time between transplantation and inclusion (model 9); for smoking (model 10); for history of cardiovascular disease (model 11); for hsCRP (model 12); for waist circumference, triglycerides, and Framingham risk factors (model 13); as well as for presence of diabetes, HbA1c, glucose, insulin, use of insulin, and use of anti-diabetic drugs (model 14) did not substantially change the association. In univariate Cox regression analyses, plasma concentrations of sPLA_2_-IIA were strongly associated with all-cause mortality (HR = 1.39 (1.17–1.64), *p* < 0.001, model 1, Table 2) as well as cardiovascular mortality (HR = 1.48 (1.18–1.85), *p* = 0.001, model 1, Table 2). However, these associations were weakened after adjustment for patient age and sex (model 2). In agreement with the findings for graft failure, these associations were no longer present after subsequent adjustments for serum creatinine (model 3), creatinine clearance (model 4), and eGFR (model 5). Furthermore, the association of sPLA_2_-IIA levels with all-cause mortality and cardiovascular mortality also disappeared after adjustment for urinary protein excretion (model 6), and adjustment for proteinuria (model 7) rendered these associations much weaker. Conversely, these associations remained after adjustments for donor age and sex (model 8); for living donor, dialysis vintage, and time between transplantation and inclusion (model 9); for smoking (model 10); for history of CVD (model 11); for waist circumference, triglycerides, and Framingham risk factors (model 13); and for presence of diabetes, HbA1c, glucose, insulin, use of insulin, and use of anti-diabetic drugs (model 14). Notably, although the increased risk of graft failure in RTRs with high sPLA_2_-IIA concentrations persisted after adjustment for hsCRP, plasma sPLA_2_-IIA levels were no longer associated with all-cause mortality and cardiovascular mortality after adjustment for this additional inflammatory marker (model 12).

**Figure 3:**
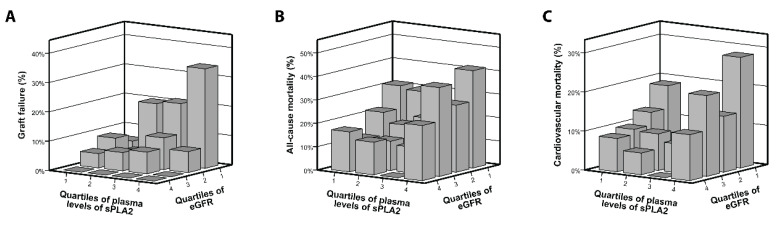
Graft failure (**A**), all-cause mortality (**B**), and cardiovascular mortality (**C**) according to quartiles of plasma levels of group IIA secretory phospholipase A_2_ (sPLA_2_) and estimated glomerular filtration rate (eGFR).

The association of circulating sPLA_2_-IIA levels with graft failure, all-cause mortality, and cardiovascular mortality appeared to be principally explained by renal allograft function. From Cox regression analysis, it cannot be judged whether this is because of confounding or represents a causal relation. To determine whether high levels of sPLA_2_-IIA are the cause or consequence of impaired kidney function, we evaluated the incidence of graft failure, all-cause mortality, and cardiovascular mortality at follow-up according to quartiles of sPLA_2_-IIA and quartiles of eGFR. In the quartile with the lowest eGFR at baseline, the incidence of graft failure gradually increased with increasing quartiles of plasma levels of sPLA_2_-IIA (quartile 1 sPLA_2_-IIA 6.3%, quartile 2 sPLA_2_-IIA 20.0%, quartile 3 sPLA_2_-IIA 21.1%, and quartile 4 sPLA_2_-IIA 34.0%; Figure 3A). In contrast, none of the patients in the quartile with the highest eGFR at baseline developed graft failure (Figure 3A). These differences were not observed for all-cause mortality (Figure 3B) and cardiovascular mortality (Figure 3C). These findings indicate that plasma sPLA_2_-IIA concentrations at baseline only seem to impact graft outcome in RTRs with the lowest baseline eGFR.

**Figure 4:**
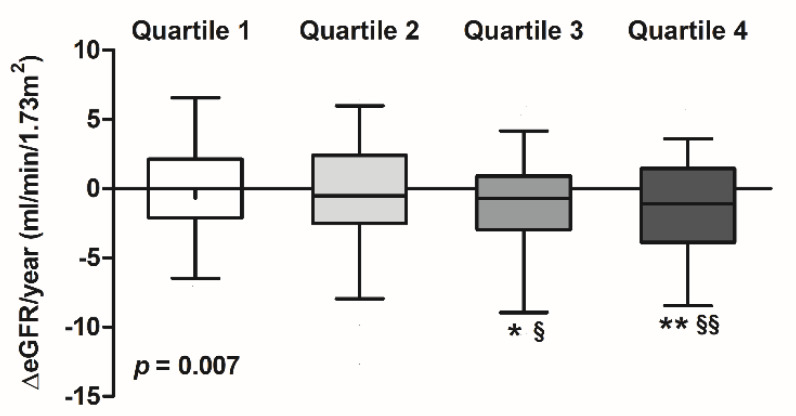
Decline in kidney function as measured by estimated glomerular filtration rate (eGFR) according to sex-stratified quartiles of plasma levels of group IIA secretory phospholipase A_2_ (sPLA_2_-IIA). Data are shown as box plots, with the middle vertical line indicating the median, the limits of the box indicating the interquartile range, and the whiskers indicating the 5th and 95th percentiles. The *p*-value represents results for the Kruskal–Wallis test. * *p* = 0.015 versus quartile 1; ** *p* = 0.005 versus quartile 1; § *p* = 0.048 versus quartile 2; §§ *p* = 0.013 versus quartile 2.

As a next step, we calculated the decline in kidney function as measured by eGFR across the sex-stratified quartiles of plasma levels of sPLA_2_-IIA. During the first three years of follow-up, kidney function declined faster in RTRs in the third quartile compared with both the first and second quartile of sPLA_2_-IIA (*p* = 0.015 and *p* = 0.048, respectively, Figure 4), and in the fourth quartile compared with both the first and second quartile of sPLA_2_-IIA (*p* = 0.005 and *p* = 0.013, respectively, Figure 4). These data suggest that elevated baseline levels of sPLA_2_-IIA prospectively associate with the rate of decline of kidney function in RTRs during follow-up.

## 4. Discussion

This study demonstrates that, in RTRs, sPLA_2_-IIA serves as a significant predictive biomarker for the occurrence of chronic graft failure, overall mortality, and specific cardiovascular disease mortality, however, it is not independent of kidney function. In addition, we show that higher baseline sPLA_2_-IIA levels accelerate the decrease in kidney function in RTRs over time. These data suggest that sPLA_2_-IIA might serve as a useful causative biomarker to assess the risk of chronic graft failure in RTRs at an early timepoint. This study further suggests that pharmacological intervention strategies to inhibit sPLA_2_-IIA might be valuable to reduce chronic graft failure.

Data derived from preclinical models as well as from clinical studies thus far indicate that sPLA_2_-IIA is a cardiovascular disease biomarker with intrinsic pro-atherosclerotic biological activities [10]. Overexpression of sPLA_2_-IIA in wild-type mice reduces circulating plasma levels of HDL by accelerating its catabolism [16,18], while in mice with transgenic expression of cholesteryl ester transfer protein (CETP), sPLA_2_-IIA activity was demonstrated to inhibit CETP [39]. In addition, sPLA_2_-IIA-modified HDL loses its anti-oxidative protective function, which had been attributed to reduced amounts of paraoxonase on such HDL [15]. On the other hand, it has been shown that sPLA_2_-IIA enhances the formation of aggregated/fused LDL particles, thereby increasing the atherogenicity of LDL [40]. In addition, macrophage as well as platelet sPLA_2_-IIA expression increase LDL oxidation [19,41], an effect that can potentially be blocked with angiotensin II type 1 receptor antagonists [42]. A potential impact of these drugs in our cohort could not be examined, as they were not used. In addition to its impact on lipoproteins, the enzymatic activity of sPLA_2_-IIA is linked to oxidative stress and inflammation. sPLA_2_-IIA cleaves phospholipids at the sn-2 position, thereby mainly liberating arachidonic acid, which is then fueled into cyclooxygenase and lipoxygenase pathways, resulting in the generation of reactive oxygen species (ROS) and pro-inflammatory mediators such as prostaglandins and leukotrienes [10]. In the vessel wall, specifically in vascular smooth muscle cells, the expression of sPLA_2_-IIA causes endothelial dysfunction [27]. Interestingly, cyclooxygenase 2 has been implicated in this process. Human sPLA_2_-IIA transgenic mice have increased vascular cyclooxygenase 2 expression and activity as well as ROS production, which can be blocked with cyclooxygenase 2-specific inhibitors; in humans, cyclooxygenase 2 activity correlated with plasma sPLA_2_-IIA levels [27]. The combination of these properties of sPLA_2_-IIA leads to an increased formation of atherosclerotic lesions in mouse models [10,15,19]. These data make it likely that sPLA_2_-IIA also accelerates the formation of transplant vasculopathy, as demonstrated in our present study. A decrease in kidney function caused by sPLA_2_-IIA is hereby a novel, previously unrecognized feature of the enzyme. Consistent with these preclinical data, prospective studies using circulating sPLA_2_-IIA levels as a biomarker for cardiovascular disease in patient populations without obvious impairment of kidney function demonstrated this phospholipase to be significantly associated with the occurrence of future cardiovascular disease events [21,24,25,26]. It would be interesting to analyze in these studies if an sPLA_2_-IIA-induced impairment in kidney function may have been a contributing factor to the outcomes, because even a small decrease in renal function is already associated with a significant effect on cardiovascular disease mortality [43].

However, despite the vast evidence for a causal involvement of sPLA_2_-IIA in the atherosclerotic disease process, the clinical development of specific sPLA_2_-IIA inhibitors has thus far not been a success story [10]. Initially, inhibitors of sPLA_2_-IIA activity were developed for use in patients with sepsis, but a respective large multicenter, double-blind, placebo-controlled clinical trial was terminated prematurely because of futility [44]. Subsequently, the sPLA_2_-IIA inhibitor varespladib was tested as anti-atherosclerotic therapeutic, but a recent double-blind, randomized, multicenter trial assessing its efficacy in patients after acute myocardial infarction also showed a negative result with respect to reduction of a composite cardiovascular outcome measure, and was also prematurely terminated.

sPLA_2_-IIA is an acute phase protein [10], as mirrored also in our study by the tight correlation with hsCRP. Thus, the pro-inflammatory pathways leading to the induction of both proteins are likely similar. However, the (patho)physiological processes caused by their respective induction appear to be different. While correcting for hsCRP in Cox regression models offsets the significant association of sPLA_2_-IIA with overall and cardiovascular-specific mortality, the association with chronic graft failure was not affected, pointing to a specific impact of sPLA_2_-IIA on transplant vasculopathy independent of a more generalized systemic inflammatory response. Therefore, another relevant topic for future research appears to be a further, more detailed delineation of the mechanism of how sPLA_2_-IIA might be causing an impairment in kidney function. Proinflammatory mediators from cyclooxygenase or lipoxygenase pathways might be such a contributing factor [10]. In addition, sPLA_2_-IIA is known to exert proinflammatory signaling via the so-called M-type receptor [10]. Moreover, this pathway could conceivably contribute to the impairment in kidney function suggested by the current study. Interestingly, antibodies directed against the M-type receptor were indicated as potentially disease causing in membranous nephropathy and are held responsible for the recurrence of the disease after renal transplantation [45]. Further insights into the sPLA_2_-IIA-related signal transduction pathways might thus allow the identification of a more specific point for therapeutic intervention into the pathway of atherosclerosis development within kidney grafts.

Potential limitations of our work also merit commenting. This study is from a single center in the North of the Netherlands, thus representing a population with a relatively homogenous as well as narrow Caucasian genetic background. Thus, replication would be required to inform if the obtained results are generalizable. In addition, although we investigated a relatively large cohort of RTRs, the number of participants is still limited, thus potentially impacting predictive power. Further, longitudinal studies as follow-up of our work would be advisable to obtain further evidence that the relationship between sPLA_2_-IIA and chronic graft failure is causative; for this purpose, also studies in preclinical models would be helpful.

In summary, the present study demonstrates that sPLA_2_-IIA is a significant predictive biomarker for the occurrence of chronic graft failure, overall mortality, and specific cardiovascular disease mortality. The association is significantly modified by kidney function measures. Inhibition of sPLA_2_-IIA or respective targets of its downstream signaling cascade might prove useful as novel therapeutics to prevent or treat chronic graft vasculopathy, a significant clinical problem thus far poorly amenable to pharmacotherapy.

## Figures and Tables

**Table 1:** Baseline characteristics according to sex-stratified quartiles of plasma levels of group IIA secretory phospholipase A_2_ (sPLA_2_-IIA).

	Sex-Stratified Quartiles of Plasma Levels of sPLA_2_-IIA				
	First (*n* = 127)	Second (*n* = 128)	Third (*n* = 129)	Fourth (*n* = 127)	*p* Value
sPLA_2_ (ng/dL)	227.2 (196.1–248.3)	321.4 (290.7–356.5)^c^	444.7 (402.0–504.3) ^c,f^	757.4 (610.1–968.2) ^c,f,i^	<0.001
***Recipient demographics***					
Age, years	50.5 ± 12.3	50.2 ± 12.7	51.8 ± 11.7	54.3 ± 10.9 ^d^	0.026
Male sex, *n* (%)	68 (54)	69 (54)	69 (54)	68 (54)	1.000
Current smoking, *n* (%)	18 (14)	19 (15)	32 (25) ^a,d^	44 (35) ^c,f^	<0.001
Previous smoking, *n* (%)	58 (46)	59 (46)	52 (40)	53 (42)	0.732
Metabolic syndrome, *n* (%)	64 (50)	78 (61)	80 (62)	70 (55)	0.067
***Body composition***					
BMI, kg/m^2^	25.4 ± 4.2	25.7 ± 4.6	26.6 ± 4.1	26.3 ± 4.4	0.117
Waist circumference men, cm	98.3 ± 11.8	98.7 ± 11.9	99.7 ± 13.1	102.7 ± 13.1	0.159
Waist circumference women, cm	90.2 ± 13.1	93.9 ± 16.9	96.3 ± 14.2	94.5 ± 14.4	0.149
***Blood pressure***					
Systolic blood pressure, mmHg	152.1 ± 23.3	149.1 ± 20.0	154.1 ± 23.4	155.9 ± 25.8	0.113
Diastolic blood pressure, mmHg	89.5 ± 9.9	88.8 ± 9.4	90.2 ± 10.3	90.1 ± 10.2	0.642
Use of ACE inhibitors, *n* (%)	41 (32)	45 (35)	48 (37)	43 (34)	0.864
Use of β-blockers, *n* (%)	79 (62)	80 (63)	80 (62)	75 (59)	0.937
Use of diuretics, *n* (%)	50 (39)	47 (37)	63 (49)	68 (54) ^a,e^	0.022
Number of anti-hypertensive drugs, *n*	2.0 (1.0–3.0)	2.0 (1.0–3.0]	2.0 (1.0–3.0]	2.0 (1.0–3.0]	0.505
***Lipids***					
Total cholesterol, mmol/L	5.5 ± 0.9	5.5 ± 0.9	5.9 ± 1.3^d^	5.7 ± 1.2	0.024
LDL-cholesterol, mmol/L	3.6 ± 0.8	3.4 ± 0.9	3.6 ± 1.2	3.6 ± 1.1	0.329
HDL-cholesterol, mmol/L	1.1 ± 0.3	1.1 ± 0.3	1.1 ± 0.3	1.1 ± 0.4	0.756
Triglycerides, mmol/L	1.6 (1.2–2.2)	1.9 (1.4–2.5) ^a^	2.2 (1.7–2.9) ^c,e^	1.9 (1.5–2.6) ^b,g^	<0.001
Use of statins, *n* (%)	55 (43)	73 (57)	67 (52)	58 (46)	0.116
***Cardiovascular disease history***					
Myocardial infarction, *n* (%)	6 (5)	12 (9)	12 (9)	15 (12)	0.260
TIA/CVA, *n* (%)	9 (7)	5 (4)	5 (4)	6 (5)	0.585
***Glucose homeostasis***					
Glucose, mmol/L	4.5 (4.1–5.0)	4.6 (4.2–5.0)	4.5 (4.1–5.0)	4.7 (4.1–5.2)	0.621
Insulin, μmol/L	10.6 (7.6–14.1)	11.2 (8.3–15.3)	12.1 (8.8–16.4)	10.2 (7.1–14.9)	0.072
HbA1c, %	6.2 (5.7–6.7)	6.2 (5.8–6.9)	6.6 (6.0–7.3) ^c,e^	6.6 (6.0–7.5) ^c,f^	<0.001
HOMA	2.1 (1.6–3.5)	2.3 (1.6–3.4)	2.5 (1.8–3.6)	2.1 (1.5–3.5)	0.227
Pre-Tx diabetes mellitus, *n* (%)	3 (2)	5 (4)	7 (5)	9 (7)	0.321
Post-Tx diabetes mellitus, *n* (%)	30 (24)	22 (17)	24 (19)	21 (17)	0.466
Use of anti-diabetic drugs, *n* (%)	20 (16)	18 (14)	19 (15)	15 (12)	0.831
Use of insulin, *n* (%)	4 (3)	9 (7)	10 (8)	11 (9)	0.306
***Inflammation***					
hsCRP, mg/L	1.4 (0.6–3.1)	1.3 (0.5–2.8)	2.8 (1.1–5.6)^c,f^	4.9 (1.8–10.0) ^c,f,h^	<0.001
***CMV status***					
CMV seropositivity, *n* (%)	92 (72)	88 (70)	94 (73)	92 (72)	0.873
***Donor demographics***					
Age, years	35.4 ± 15.9	37.7 ± 16.1	35.9 ± 15.2	39.0 ± 15.0	0.219
Male sex, *n* (%)	74 (58)	70 (55)	73 (57)	66 (52)	0.743
Number of HLA mismatches	1 (0–2)	2 (0–3)	2 (1–3)	2 (0–3)	0.409
***(Pre)transplant history***					
Dialysis vintage, months	28.0 (10.0–48.0)	25.5 (13.5–46.0)	30.0 (18.0–47.0)	26.0 (11.5–52.0)	0.477
Time between Tx and inclusion, years	6.6 (3.4–11.3)	5.7 (2.6–11.1)	6.1 (3.4–11.4)	6.5 (2.2–12.1)	0.519
***Transplantation type***					
Living kidney donor, *n* (%)	18 (14)	19 (15)	17 (13)	11 (9)	0.445
Postmortem donor, *n* (%)	109 (86)	109 (85)	112 (87)	116 (91)	0.445
Acute rejection, *n* (%)	52 (41)	57 (45)	53 (41)	57 (45)	0.870
***Immunosuppression***					
Daily prednisolone, mg/d	10.0 (7.5–10.0)	10.0 (8.8–10.0)	10.0 (7.5–10.0)	10.0 (7.5–10.0)	0.211
Calcineurin inhibitors, *n* (%)	96 (76)	109 (85)	106 (82)	94 (74)	0.088
Proliferation inhibitors, *n* (%)	95 (75)	96 (75)	92 (71)	91 (72)	0.858
***Renal allograft function***					
Serum creatinine, μmol/L	121.0 (103.5–146.0)	129.0 (111.0–152.0) ^a^	139.0 (116.0–172.0) ^c,d^	148.0 (122.5–215.5) ^c,f,g^	<0.001
Creatinine clearance, mL/min	67.2 ± 21.4	65.1 ± 20.8	59.5 ± 21.0 ^a^	53.7 ± 23.0 ^c,f^	<0.001
eGFR, mL/min/1.73m^2^	53.2 ± 14.2	49.6 ± 14.3	45.2 ± 15.7 ^c^	39.6 ± 16.8 ^c,f,g^	<0.001
Urinary protein excretion, g/24h	0.2 (0.0–0.4)	0.2 (0.0–0.4)	0.2 (0.0–0.5)	0.3 (0.2-–0.7) ^b,e^	0.002
Proteinuria ≥0.5 g/24h, *n* (%)	31 (24)	28 (22)	36 (28)	51 (40) ^b,e,g^	0.007

Data are presented as mean ± standard deviation (SD) or *n* (%), and data with a skewed distribution are presented as median (25th–75th percentile). Differences were tested with one-way analysis of variance (ANOVA) followed by Bonferroni post hoc test or Kruskal–Wallis test followed by Mann–Whitney U test for continuous variables, and χ^2^ test for categorical data. ACE, angiotensin-converting enzyme; BMI, body mass index; CVA, cerebrovascular event; CMV, cytomegalovirus; eGFR, estimated glomerular filtration rate; HDL, high-density lipoprotein; HOMA, homeostatic model assessment; hsCRP, high-sensitivity C-reactive protein; LDL, low-density lipoprotein; sPLA_2_-IIA, group IIA secretory phospholipase A_2_; TIA, transient ischemic attack; Tx, transplantation. ^a^ *p* < 0.05 compared to the first quartile; ^b^ *p* < 0.01 compared to the first quartile; ^c^ *p* < 0.001 compared to the first quartile; ^d^ *p* < 0.05 compared to the second quartile; ^e^ *p* < 0.01 compared to the second quartile; ^f^ *p* < 0.001 compared to the second quartile; ^g^ *p* < 0.05 compared to the third quartile; ^h^ *p* < 0.01 compared to the third quartile; ^i^ *p* < 0.001 compared to the third quartile.

**Table 2:** Univariate and multivariate Cox regression analyses for graft failure, all-cause mortality, and cardiovascular mortality by plasma levels of group IIA secretory phospholipase A_2_ (sPLA_2_-IIA).

	Graft Failure		All-Cause Mortality		Cardiovascular Mortality	
	(47 Events)		(110 Events)		(57 Events)	
	HR (95% CI) Per 1 SD Increase	*p* Value	HR (95% CI) Per 1 SD Increase	*p* Value	HR (95% CI) Per 1 SD increase	*p* Value
Model 1	1.42 (1.11–1.83)	0.006	1.39 (1.17–1.64)	<0.001	1.48 (1.18–1.85)	0.001
Model 2	1.66 (1.26–2.19)	<0.001	1.21 (1.02–1.43)	0.027	1.26 (1.01–1.58)	0.040
Model 3	0.91 (0.68–1.21)	0.519	1.08 (0.90–1.30)	0.418	1.15 (0.90–1.47)	0.278
Model 4	1.09 (0.85–1.41)	0.492	1.02 (0.86–1.20)	0.855	1.04 (0.83–1.30)	0.728
Model 5	1.00 (0.75–1.33)	0.989	1.09 (0.91–1.30)	0.371	1.15 (0.90–1.46)	0.263
Model 6	1.58 (1.18–2.12)	0.002	1.17 (0.99–1.39)	0.069	1.23 (0.98–1.55)	0.074
Model 7	1.56 (1.16–2.10)	0.003	1.20 (1.01–1.42)	0.041	1.25 (1.00–1.57)	0.055
Model 8	1.57 (1.19–2.06)	0.001	1.27 (1.07–1.51)	0.007	1.30 (1.03–1.64)	0.028
Model 9	1.69 (1.28–2.23)	<0.001	1.21 (1.02–1.43)	0.029	1.27 (1.01–1.59)	0.039
Model 10	1.63 (1.23–2.17)	0.001	1.21 (1.02–1.43)	0.026	1.27 (1.02–1.58)	0.036
Model 11	1.63 (1.24–2.16)	0.001	1.23 (1.04–1.46)	0.018	1.27 (1.01–1.60)	0.039
Model 12	1.68 (1.22–2.31)	0.001	1.11 (0.92–1.34)	0.282	1.11 (0.86–1.43)	0.421
Model 13	1.51 (1.13–2.03)	0.006	1.28 (1.08–1.53)	0.006	1.39 (1.10–1.76)	0.005
Model 14	1.64 (1.22–2.21)	0.001	1.27 (1.06–1.53)	0.010	1.31 (1.01–1.70)	0.040

Model 1: crude; model 2: model 1 + adjustments for recipient age and sex; model 3: model 2 + adjustment for serum creatinine; model 4: model 2 + adjustment for creatinine clearance; model 5: model 2 + adjustment for eGFR; model 6: model 2 + adjustment for urinary protein excretion; model 7: model 2 + adjustment for proteinuria; model 8: model 2 + adjustments for donor age and sex; model 9: model 2 + adjustments for living donor, dialysis vintage, and time between transplantation and inclusion; model 10: model 2 + adjustments for smoking; model 11: model 2 + adjustment for history of cardiovascular disease; model 12: model 2 + adjustment for log hsCRP; model 13: model 2 + adjustments for waist circumference, log triglycerides, and Framingham risk factors (systolic blood pressure, presence of diabetes, LDL-cholesterol, HDL-cholesterol, smoking); model 14: model 2 + adjustments for presence of diabetes, Hb1Ac, glucose, insulin, use of insulin, and use of anti-diabetic drugs. HR, hazard ratio; CI, confidence interval.

## Supplementary Materials

The following are available online at https://www.mdpi.com/2077-0383/9/5/1282/s1, Table S1: Clinical and biochemical characteristics of the end-stage renal disease (ESRD) patients and controls investigated.
