# Supplementary material for: Group IIA Secretory Phospholipase A2 Predicts Graft Failure and Mortality in Renal Transplant Recipients by Mediating Decreased Kidney Function
Source: J Clin Med. 2020 Apr 29;9(5):1282. doi: 10.3390/jcm9051282 (PMC7288094; doi:10.3390/jcm9051282)
Supplement: Supplementary file 1 [file jcm-09-01282-s001.pdf]

Supplemental tables

Supplemental table I. Clinical and biochemical characteristics of the end-stage renal disease (ESRD) patients and controls investigated.

	ESRD (n=60)	Controls (n=30)	*P* value
Age (years)	49 (25-61)	50 (30-64)	n.s.
Sex (male/female)	32/28	17/13	n.s.
Body weight (kg)	76 (56-86)	67 (58-79)	<0.05
Duration of dialysis (months)	41 (24-88)	0	
Serum creatinine (mg/dl)	7.2 (5.4-9.8)	1.0 (0.7-1.2)	<0.001
Blood urea nitrogen (mg/dl)	28 (22-42)	15 (10-17)	<0.001
Total protein (g/l)	65 (50-83)	70 (67-74)	n.s.
Cholesterol (mg/dl)	251 (184-298)	204 (166-234)	<0.05
CRP (mg/l)	5.6 (1.8-8.7)	1.2 (0.7-1.6)	<0.001
Smoking	10 (16)	0 (0)	0.018
Diabetes	28 (47)	0 (0)	<0.001
Coronary heart disease	21 (35)	0 (0)	<0.001

Data are presented as median (range) or total numbers (%).
